# Supplementary material for: Neuronal correlates of endogenous selective attention in the endbrain of crows
Source: Commun Biol. 2025 Mar 21;8:470. doi: 10.1038/s42003-025-07914-2 (PMC11928645; doi:10.1038/s42003-025-07914-2)
Supplement: Supplementary file 2 — Supplementary Material [file 42003_2025_7914_MOESM2_ESM.pdf]

Supplementary Materials for

Neuronal correlates of endogenous selective attention in the endbrain of crows

Lukas A. Hahn, Erica Fongaro, Jonas Rose*

*Corresponding author. Email: *jonas.rose@rub.de*

This PDF file includes:

Figs. S 1 to S 2

Tables S1 to S 5

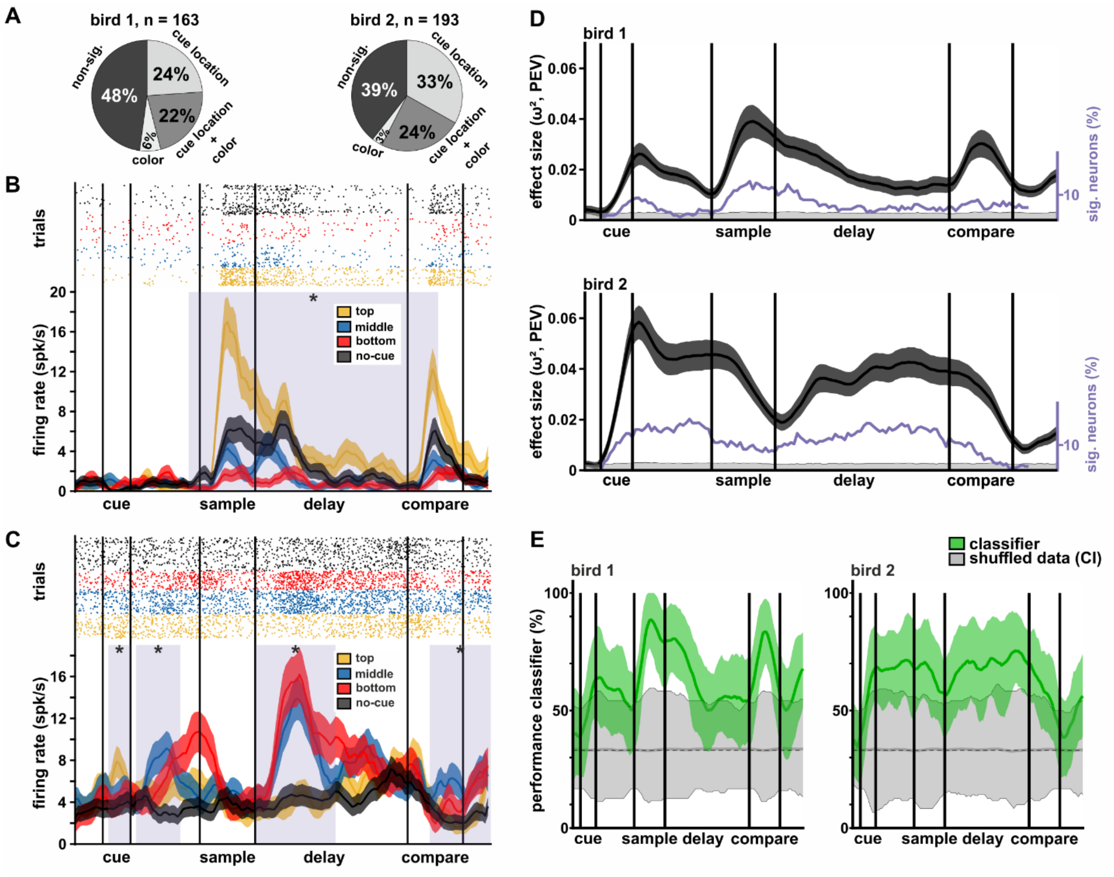

Fig. S 1. Pre-cue location results of each individual animal. (**A**) Overview of proportions of significant neurons (percentages rounded to the nearest integer), per bird. (**B**) Example neuron showing substantial differentiation of firing rates for different cue locations, during the sample phase. Upper part: dot-raster plot of neuronal spiking, each line represents an individual spike, sorted and color coded based on location of the cue. Trials without a cue in black. Lower part: Peri-stimulus time histogram of firing rates in different trial conditions. Top, middle, and bottom refer to the location of the cue during the cue-phase. Solid line indicates the mean firing rate, shaded area around the line indicates the standard error of the mean. Purple shading indicates the period when firing rates between the different cue locations were significantly different. (**C**) Same as in (B) for another example neuron. (**D**) Mean PEV (shaded area indicates standard error of the mean) of all neurons, per bird. In purple: percentage of neurons with significant amount of information at the respective timepoint (right axis). Grey shaded area indicates PEV of the randomized data set. (**E**) Results of a SVM classifier, along the behavioral protocol, per bird. Time bins (200 ms, advanced in 20 ms steps) for testing and training were the same. Based on neuronal activity of neurons with significant activity for the factor location. Grey shading indicates results of the classifier based on shuffled data (inner 95 % of null distribution).

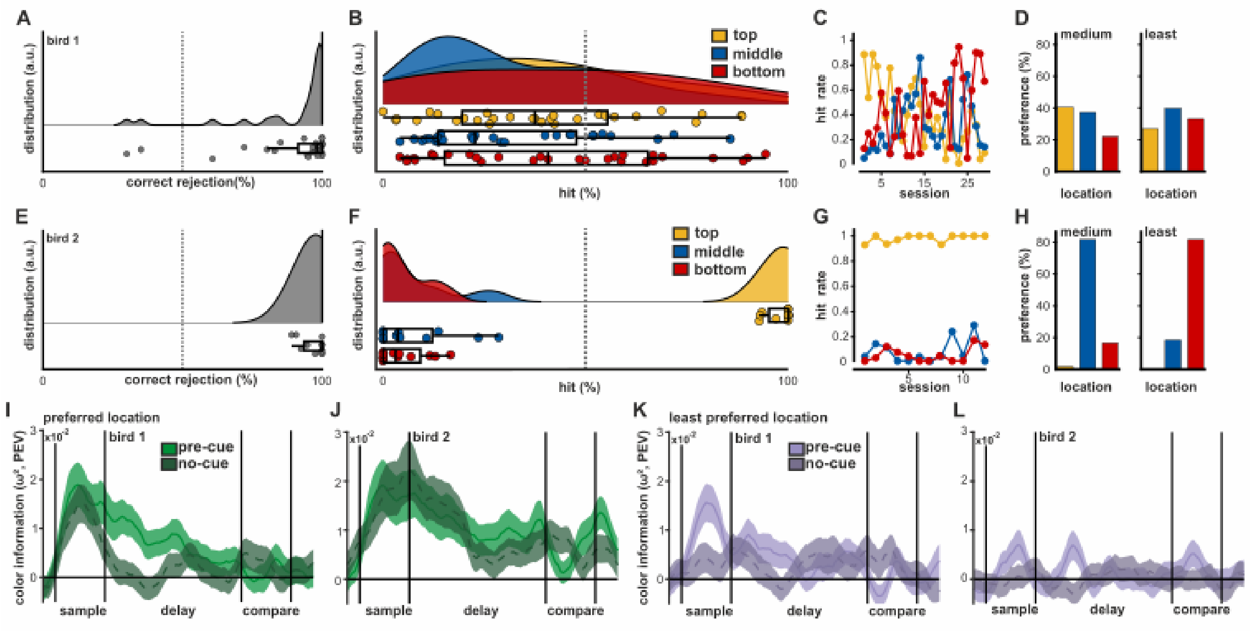

Fig. S 2. No-cue trials behavioral results. Bird 1: (**A**) Correct rejections in no-cue trials. (**B**) Hit performance in no-cue trials (over all sessions). (**C**) Hit performance per session. (**D**) Proportions of medium and least preferred locations. Bird 2 (**E-H**): Same as in (A-D). (**I**) Color information at preferred location of bird 1. Solid line: Color information throughout the trial at the preferred location when the cue was presented at that location. Dashed line: Color information when there was no cue. Lines indicate the mean; shaded areas are the standard error of the mean. (**J**) Same as (I) for bird 2. (**K & L**) Same as in (I & J) for the least preferred location.

Table S 1. Statistical results of data shown in Fig. 4C. Wilcoxon sign-rank test of change of information depending on cue, against no-change ($\Delta = 0$). Significant results (based on Bonferroni corrected alpha) are highlighted. Results of a Bayesian one sample Wilcoxon signed rank test on the same data (default Cauchy prior, with 0.707 scale). Bayes factor for the alternative hypothesis that population has median > 0 (BF_{+0}), against the null hypothesis that it has not. C.I. denotes the credibility interval of effect sizes. Classification of evidence following van Doorn et al., (2021)[100].

	**bird 1**						
**phase**	**z-stat.**	**p-val.**	**Coh. d**	**BF_{+0}**	**W**	**C.I.**	**Evidence for H_A**
early sample	z = 3.2263	.0013	0.3198	180.085	8139	[0.156 0.471]	Very strong
late sample	z = 2.1321	.0330	0.2535	8.661	7061	[0.069 0.388]	Moderate
delay 1	z = 2.3462	.0190	0.2540	13.936	7449	[0.078 0.393]	Strong
delay 2	z = 1.8826	.0598	0.1990	2.109	7187	[0.032 0.339]	Weak
delay 3	z = 2.4745	.0133	0.2425	7.715	7249	[0.069 0.394]	Moderate
delay 4	z = -0.1829	.8549	0.0673	0.152	5565	[0.004 0.213]	No. Moderate evidence for H_0
delay 5	z = 1.3689	.1710	0.1405	0.608	6642	[0.015 0.284]	Weak evidence for H_0
early compare	z = -0.4484	.6539	0.0133	0.081	5794	[0.002 0.176]	No. Strong evidence for H_0
	**bird 2**						
	**z-stat.**	**p-val.**	**Cohen's d**	**BF_{+0}**	**W**	**C.I.**	**Evidence for H_A**
early sample	z = 2.6867	.0072	0.2447	36.104	10562	[0.105 0.390]	Very strong
late sample	z = 1.0384	.2991	0.1385	0.595	9459	[0.013 0.260]	No. Weak evidence for H_0
delay 1	z = 1.8632	.0624	0.2106	3.813	9755	[0.048 0.324]	Moderate
delay 2	z = 2.6216	.0088	0.2589	17.728	10086	[0.090 0.376]	Strong
delay 3	z = 1.4871	.1370	0.1420	0.962	9186	[0.021 0.284]	No. Weak evidence for H_0

delay 4	z = 0.4134	.6793	0.0839	0.235	8342	[0.006 0.221]	No. Moderate evidence for H_0
delay 5	z = -1.0945	.2737	< 0.0001	0.063	7212	[0.002 0.154]	No. Strong evidence for H_0
early compare	z = 0.6485	.5166	0.1095	0.374	8599	[0.010 0.244]	No. Moderate evidence for H_0

Table S 2. Statistical results of data shown in Fig. 4D. Wilcoxon sign-rank test of change of information depending on cue, against no-change (Δ = 0). Significant results (based on Bonferroni corrected alpha) are highlighted.

	**bird 1**			**bird 2**		
**phase**	**z-statistics**	**p-val.**	**Cohen's d**	**z-statistics**	**p-val.**	**Cohen's d**
early sample	z = 2.3267	.0200	0.2138	z = 2.4543	.01412	0.2020
late sample	z = 2.2365	.0253	0.1977	z = 0.6994	.4843	0.0509
delay 1	z = 3.2403	.0012	0.2354	z = 1.7403	.08181	0.1509
delay 2	z = 2.2229	.0262	0.1655	z = 1.4821	.1383	0.1250
delay 3	z = 1.9109	.0560	0.1793	z = 1.8440	.0652	0.1305
delay 4	z = -0.2680	.7887	0.0207	z = -0.1894	.8498	0.0006
delay 5	z = 1.1582	.2468	0.1149	z = -1.3615	.1733	-0.0998
early compare	z = -0.6324	.5271	-0.0410	z = 0.5060	.6129	0.0443

Table S 3. Statistical results of data shown in Fig. 4E. Wilcoxon sign-rank test of change of information depending on cue, against no-change (Δ = 0). Significant results (based on Bonferroni corrected alpha) are highlighted.

	**bird 1**			**bird 2**		
**phase**	**z-statistics**	**p-val.**	**Cohen's d**	**z-statistics**	**p-val.**	**Cohen's d**
early sample	z = -4.2906	<.0001	-0.3589	z = -3.8041	0.0001	-0.2602
late sample	z = -2.4650	.0137	-0.2580	z = -1.9594	0.0501	-0.1807
delay 1	z = -2.3115	.0208	-0.2369	z = -2.8254	0.0047	-0.2199
delay 2	z = -2.0146	.0439	-0.1948	z = -3.5862	0.0003	-0.3114
delay 3	z = -3.5497	.0004	-0.2463	z = -1.1346	0.2566	-0.0914
delay 4	z = -0.6112	.5411	-0.1174	z = -1.7022	0.0887	-0.1593
delay 5	z = -2.0925	.0364	-0.1107	z = -0.87995	0.3789	-0.1090
early compare	z = -1.4397	.1500	-0.0647	z = -1.6573	0.0975	-0.1380

Table S 4. Statistical results of cross trial type classification of bird 1. X^2-test on equality of classification of no-cue trials by the classifier trained on pre-cue trials, also shown in Fig. 5 C area plot.

	chi2	d.f.	p-value		chi2	d.f.	p-value		chi2	d.f.	p-value		chi2	d.f.	p-value
**sample**	18.65	2	<0.0001	**early delay**	2.15	2	0.3413	**late delay**	3.2	2	0.2019	**compare**	9.8	2	0.0074
	36.05	2	<0.0001		3.2	2	0.2019		8.45	2	0.0146		5.15	2	0.0762
	8.45	2	0.0146		2.45	2	0.2938		4.55	2	0.1028		6.65	2	0.0360
	2.6	2	0.2725		1.55	2	0.4607		5.15	2	0.0762		18.05	2	0.0001
	4.85	2	0.0885		0.65	2	0.7225		9.05	2	0.0108		2.45	2	0.2938
	5.15	2	0.0762		0.65	2	0.7225		18.65	2	<0.0001		5.15	2	0.0762
	3.05	2	0.2176		2.6	2	0.2725		21.35	2	<0.0001		0.8	2	0.6703
	6.95	2	0.0310		0.8	2	0.6703		10.55	2	0.0051		7.55	2	0.0229
	2.45	2	0.2938		3.65	2	0.1612		9.05	2	0.0108		21.35	2	<0.0001
	1.4	2	0.4966		5.15	2	0.0762		12.35	2	0.0021		6.65	2	0.0360
	0.65	2	0.7225		6.95	2	0.0310		18.2	2	0.0001		7.55	2	0.0229
	0.2	2	0.9048		7.55	2	0.0229		11.45	2	0.0033		2.6	2	0.2725
	1.4	2	0.4966		1.55	2	0.4607		9.65	2	0.0080		6.05	2	0.0486
	1.25	2	0.5353		2.45	2	0.2938		8.15	2	0.0170		6.2	2	0.0450
	0.2	2	0.9048		0.65	2	0.7225		15.05	2	0.0005		7.55	2	0.0229
	2.6	2	0.2725		2.15	2	0.3413		8.75	2	0.0126		13.55	2	0.0011
	9.8	2	0.0074		4.55	2	0.1028		11.15	2	0.0038		12.35	2	0.0021
	8.75	2	0.0126		8.45	2	0.0146		7.4	2	0.0247		8.15	2	0.0170
	6.65	2	0.0360		8.6	2	0.0136		12.2	2	0.0022		13.55	2	0.0011
	7.85	2	0.0197		7.4	2	0.0247		17.15	2	0.0002		9.95	2	0.0069
	6.65	2	0.0360		1.4	2	0.4966		11.45	2	0.0033		18.2	2	0.0001
					6.05	2	0.0486		28.55	2	<0.0001				
					0.95	2	0.6219		18.2	2	0.0001				
					3.65	2	0.1612		1.55	2	0.4607				
					18.05	2	0.0001		18.65	2	<0.0001				
					8.45	2	0.0146		0.35	2	0.8395				
					10.85	2	0.0044		6.95	2	0.0310				

Table S 5. Statistical results of cross trial type classification of bird 2. X²-test on equality of classification of no-cue trials by the classifier trained on pre-cue trials, also shown in Fig. 5 D area plot.

	chi2	d.f.	p-value		chi2	d.f.	p-value		chi2	d.f.	p-value		chi2	d.f.	p-value
**sample**	63.05	2	<.0001	**early delay**	32.6	2	<.0001	**late delay**	8.15	2	0.0170	**compare**	17.45	2	0.0002
	43.55	2	<.0001		24.8	2	<.0001		9.95	2	0.0069		14.45	2	0.0007
	36.05	2	<.0001		17.15	2	0.0002		14.6	2	0.0007		18.2	2	0.0001
	36.05	2	<.0001		17.15	2	0.0002		12.95	2	0.0015		12.95	2	0.0015
	36.95	2	<.0001		24.8	2	<.0001		18.95	2	<.0001		16.55	2	0.0003
	24.05	2	<.0001		15.05	2	0.0005		9.95	2	0.0069		24.05	2	<.0001
	21.65	2	<.0001		11.45	2	0.0033		16.55	2	0.0003		63.35	2	<.0001
	30.65	2	<.0001		21.35	2	<.0001		16.85	2	0.0002		53.75	2	<.0001
	20.15	2	<.0001		7.85	2	0.0197		18.35	2	0.0001		58.4	2	<.0001
	57.95	2	<.0001		63.35	2	<.0001		15.8	2	0.0004		53.15	2	<.0001
	48.2	2	<.0001		48.05	2	<.0001		10.4	2	0.0055		53.15	2	<.0001
	52.85	2	<.0001		12.35	2	0.0021		15.05	2	0.0005		43.85	2	<.0001
	25.4	2	<.0001		20.15	2	<.0001		9.8	2	0.0074		48.65	2	<.0001
	17.15	2	0.0002		30.65	2	<.0001		16.85	2	0.0002		48.2	2	<.0001
	18.05	2	0.0001		18.05	2	0.0001		21.65	2	<.0001		35.45	2	<.0001
	24.05	2	<.0001		43.85	2	<.0001		25.4	2	<.0001		25.55	2	<.0001
	57.8	2	<.0001		57.8	2	<.0001		14.6	2	0.0007		26.6	2	<.0001
	57.95	2	<.0001		53.15	2	<.0001		14.45	2	0.0007		27.35	2	<.0001
	26.6	2	<.0001		63.05	2	<.0001		12.95	2	0.0015		48.65	2	<.0001
	38.15	2	<.0001		13.85	2	0.0010		16.85	2	0.0002		43.55	2	<.0001
	48.2	2	<.0001		14.6	2	0.0007		18.35	2	0.0001		48.2	2	<.0001
					18.2	2	0.0001		16.55	2	0.0003				
					11.15	2	0.0038		12.35	2	0.0021				
					11.15	2	0.0038		12.05	2	0.0024				
					9.8	2	0.0074		24.05	2	<.0001				
					12.05	2	0.0024		22.4	2	<.0001				
					9.95	2	0.0069		15.05	2	0.0005				
